# Sono‐Piezo Dynamic Therapy: Utilizing Piezoelectric Materials as Sonosensitizer for Sonodynamic Therapy

**DOI:** 10.1002/advs.202417439

**Published:** 2025-02-08

**Authors:** Zhiguang Chen, Liang Sang, Yanjun Liu, ZhiQun Bai

**Affiliations:** ^1^ Department of Ultrasound The First Hospital of China Medical University No. 155, Nanjing North Street, Heping District Shenyang Liaoning 110001 China

**Keywords:** piezoelectric effect, piezoelectric materials, sonodynamic therapy, sono‐piezo dynamic therapy, sonosensitizer

## Abstract

Sonodynamic therapy (SDT) represents a promising approach for cancer treatment. Compared to photodynamic therapy, SDT offers increased penetration depth and higher precision. However, the practical application of SDT is constrained by the low water solubility, poor tumor specificity, and metabolic susceptibility of most sonosensitizers. Recent research has explored the use of piezoelectric materials as sonosensitizers in cancer treatment and inhibition of bacterial growth. Upon ultrasound excitation, the separation of electron–hole (e^−^–h^+^) pairs occurs within the piezoelectric material. By improving the crystal structure of the material or incorporating other nanoparticles to prevent rapid recombination of e^−^–h^+^ pairs, the piezoelectric material accumulates charges in the conduction band and valence band, achieving the redox potential of O_2_/·O_2_^−^. This enables the piezoelectric material to serve as a sonosensitizer, leading to the concept termed Sono‐Piezo Dynamic Therapy (SPDT). This review aims to define the concept of SPDT, provide a systematic overview of the historical development of piezoelectric materials in the application of SDT, and elucidate the potential mechanisms by which piezoelectric materials act as sonosensitizers. Importantly, various piezoelectric materials will be discussed in terms of their feasibility, advantages, and disadvantages as sonosensitizers, offering new perspectives for identifying potential sonosensitizers.

## Graphical abstract

SPDT refers to the separation of e^−^–h^+^ pairs within piezoelectric materials under the action of ultrasound, leading to the accumulation of charges in the conduction band and valence band of the piezoelectric materials, thereby achieving the redox potential of O_2_/O_2_^−^ and realizing the purpose of using piezoelectric materials as sonosensitizers.

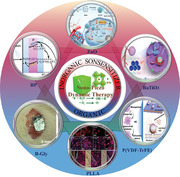

## 1 Introduction

In 1989, Umemura et al.^[^ ^1^ ^]^ and their team made a pivotal discovery: the presence of hematoporphyrin in tumor cells could substantially reduce their activity when stimulated by ultrasound. This observation laid the groundwork for the proposition of Sonodynamic Therapy (SDT) in 1992.^[^ ^2^ ^]^ The mechanism underlying SDT remains controversial, with early theories attributing its cytotoxicity to the generation of singlet oxygen (^1^O_2_).^[^ ^3^ ^]^ However, as research progressed, scholars proposed that the biological effects of SDT are based on the cavitation phenomenon induced by ultrasound. Cavitation involves the formation of microbubbles at the source within the medium under ultrasound action. Low‐intensity ultrasound stimulation only produces stable cavitation, which does not compromise the stability of the cell membrane and, consequently, cell activity. Only in synergy with sonosensitizers does SDT impact cell activity by lowering the energy threshold required for cell membrane mechanical damage under ultrasound stimulation.^[^ ^4^, ^5^, ^6^, ^7^, ^8^ ^]^ The three fundamental components of SDT are sonosensitizers, ultrasound, and oxygen. A wide range of sonosensitizers has been developed for SDT research. These can be broadly classified into two categories: organic sonosensitizers^[^ ^9^ ^]^ and inorganic sonosensitizers.^[^ ^10^ ^]^ Organic sonosensitizers encompass porphyrins and phthalocyanines,^[^ ^11^ ^]^ xanthene compounds,^[^ ^12^ ^]^ phenothiazine compounds,^[^ ^13^ ^]^ fluoroquinolone antibiotics,^[^ ^14^ ^]^ and natural products.^[^ ^15^ ^]^ Inorganic sonosensitizers encompass a diverse range of nanomaterials, including titanium dioxide (TiO_2_) nanomaterials,^[^ ^16^ ^]^ silicon and carbon nanomaterials,^[^ ^17^ ^]^ zirconium oxide,^[^ ^18^ ^]^ metal–organic frameworks,^[^ ^19^ ^]^ barium titanate (BaTiO_3_),^[^ ^20^ ^]^ zinc oxide (ZnO),^[^ ^21^ ^]^ and various other metallic nanoparticles. Additionally, non‐metallic nanoparticles such as black phosphorus (BP) have also been explored in this context.^[^ ^22^ ^]^

**Figure 1:**
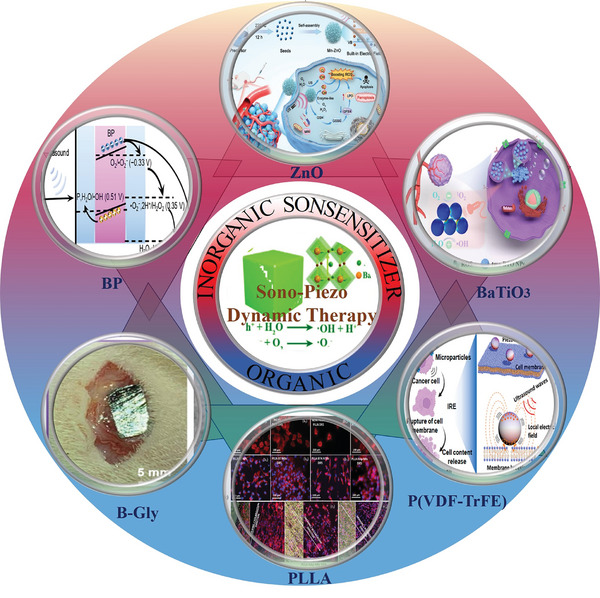
Related applications of SPDT^[^ ^60^, ^72^, ^77^, ^93^, ^103^, ^126^, ^151^ ^]^ Copyright 2023 Wiley‐VCH, Copyright 2020 WILEY‐VCH, Copyright 2023 The Authors, Copyright 2023 American Chemical Society, Copyright 2021 The Authors, Copyright 2020, American Chemical Society, Copyright 2023 Wiley‐VCH.

Among the sonosensitizers, a subset of materials exhibiting ferroelectricity and/or piezoelectricity, known as piezoelectric materials, stands out. Examples include BP,^[^ ^22^ ^]^ ZnO,^[^ ^21^ ^]^ BaTiO_3_,^[^ ^20^ ^]^ and so on. When piezoelectric materials function as sonosensitizers, ultrasound stimulation leads to the separation of electron–hole (e^−^‐h^+^) pairs occurs within the piezoelectric materials, generating an internal electric field. The resultant electrons and holes migrate to their respective opposite surfaces, producing a substantial quantity of reactive oxygen species (ROS) through redox reactions. This process thereby facilitates SDT for tumors. Furthermore, the charges accumulated on the surface of the piezoelectric materials can exert a direct effect on cancer cells, influencing the cell membrane potential. Notably, the voltage induced by the sono‐piezoelectric effect can reach up to 2.9V, sufficient to depolarize both the mitochondrial membrane and the plasma membrane, ultimately enhancing the efficacy of SDT sensitized by the piezoelectric effect.^[^ ^19^ ^]^ The application of piezoelectric materials in tumor treatment has a long history, yet the underlying therapeutic mechanisms were previously not fully elucidated. Recent studies have, however, revealed that the piezoelectric effect induced by piezoelectric materials under ultrasound stimulation enhances the sensitivity of SDT. This novel treatment modality is termed Sono‐Piezo Dynamic Therapy (SPDT).^[^ ^19^, ^23^ ^]^ SPDT is regarded as a highly promising sonodynamic therapy, as illustrated in **Figure** **1**. In this study, we aim to clearly define the concept of SPDT, provide a systematic review of the historical development of piezoelectric materials in SDT applications, and elucidate the potential mechanisms of piezoelectric materials as sonosensitizers. Additionally, we will enumerate different piezoelectric materials in terms of their feasibility, advantages, and disadvantages as sonosensitizers, offering new insights for the identification of potential sonosensitizers.

## 2 Mechanism of SDT

**Figure 2:**
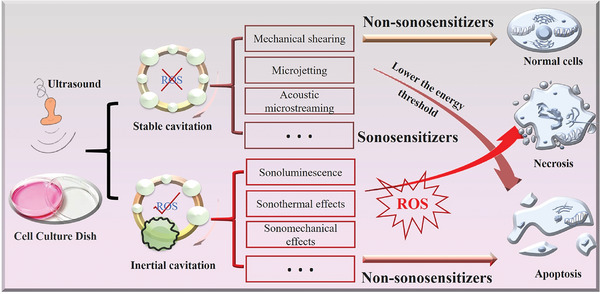
The mechanism of SDT.

The mechanism of SDT has been a subject of ongoing debate since its inception. Currently, research primarily focuses on the ultrasonic cavitation effect, which can be further classified into stable cavitation and inertial cavitation.^[^ ^24^ ^]^ Under ultrasound exposure, pressure fluctuations in the liquid medium lead to the formation of microbubbles. Some microbubbles continuously grow and rupture, a process known as inertial cavitation, while others cyclically expand and contract, termed stable cavitation.^[^ ^25^ ^]^ Stable cavitation generates mechanical shearing, microjetting, and acoustic microstreaming in the surrounding medium, but it does not produce free radicals within the microbubbles, unlike inertial cavitation.^[^ ^26^ ^]^ The energy produced by stable cavitation is insufficient to disrupt the stability of cell membranes. To induce apoptosis, the assistance of sonosensitizers is required to lower the energy threshold needed for cell membrane mechanical damage, thereby enabling stable cavitation to cause localized cell apoptosis in synergy with sensitizer agents.^[^ ^4^, ^26^ ^]^ Conversely, inertial cavitation can lead to temperatures exceeding 10000 K and pressures surpassing 81 MPa in the target area, increasing cell membrane permeability, disrupting the cell cytoskeleton and membrane structure, and accelerating cell necrosis.^[^ ^27^, ^28^ ^]^ However, the damage caused by microbubble rupture is relatively minor and may not achieve the desired therapeutic effect.^[^ ^29^, ^30^ ^]^ By incorporating sonosensitizers, SDT can exploit the extremely high temperatures and pressures induced by inertial cavitation to mediate sonoluminescence (SL), sono‐thermal effects, and sono‐mechanical effects. This allows for the generation of sufficient ROS to induce cell damage.^[^ ^31^ ^]^ Therefore, the mechanism of SDT can be broadly categorized into non‐ROS‐dependent cell damage induced by stable cavitation and ROS‐dependent cell apoptosis induced by inertial cavitation (**Figure** **2**).

Tumors constitute an evolving and intricate ecosystem characterized by significant heterogeneity.^[^ ^32^ ^]^ Traditional cancer therapies face challenges such as low drug delivery efficiency and substantial side effects. The application of adaptive nanomaterials holds the potential to enhance the effectiveness of cancer treatment.^[^ ^33^ ^]^ SDT offers unparalleled advantages in tumor treatment due to its deep penetration depth and toxicity that is not exclusively reliant on ROS. Notably, the delivery of sonosensitizers to deep‐seated tumors via nanodrug delivery systems addresses the challenges associated with treating such tumors.^[^ ^4^ ^]^ Sonosensitizers are the cornerstone of SDT, and studies have demonstrated that metal–organic frameworks (MOFs) are promising sonosensitizers owing to their rapid ROS‐generating linker‐metal charge transfer mechanism and porous structures that mitigate self‐quenching, thereby enhancing ROS production efficiency.^[^ ^34^ ^]^ MOF‐enhanced SDT has exhibited promising results in inducing apoptosis and inhibiting tumor growth; however, preclinical studies on tumor elimination remain far from satisfactory. Consequently, SDT frequently involves combination therapies, such as those combined with chemotherapy, phototherapy, gene therapy, and immunotherapy.^[^ ^35^, ^36^ ^]^ Furthermore, the optimization and innovation of sonosensitizers, particularly the development of multifunctional sonosensitizers, remain pivotal research areas and hotspots.^[^ ^9^, ^37^, ^38^ ^]^

## 3 Sonosensitizers and Piezoelectric Materials

**Figure 3:**
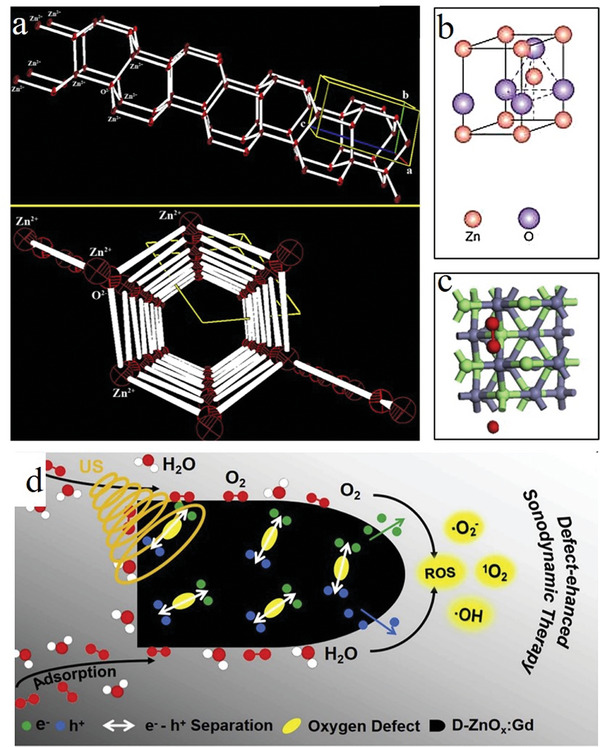
Structural schematics of ZnO as both a piezoelectric material and an ultrasound sensitizer are presented below, a) Two distinct stereographic views of the hexagonal wurtzite structure of ZnO, highlighting the tetrahedral coordination of Zn–O^[^ ^44^ ^]^ Copyright 2019 Elsevier B.V. b) Molecular structure schematic of ZnO as a piezoelectric material^[^ ^46^ ^]^ Copyright 2009 Elsevier B.V. c) Molecular structure schematic of ZnO when employed as an ultrasound sensitizer^[^ ^21^ ^]^ Copyright 2020 Elsevier Ltd. d) Schematic representation of D‐ZnOx:Gd as an ultrasound sensitizer, generating ROS^[^ ^21^ ^]^ Copyright 2020 Elsevier Ltd.

In recent years, piezoelectric materials have garnered considerable attention owing to their remarkable mechanical and electrical properties. Piezoelectric materials are substances that exhibit a charge output on their surfaces upon the application of mechanical stress. The magnitude of this charge output is directly proportional to the applied stress, a phenomenon known as the piezoelectric effect.^[^ ^39^ ^]^ The core of the piezoelectric effect lies in the conversion between mechanical energy and electrical energy. Specifically, when mechanical energy acts on a piezoelectric material, resulting in the generation of electrical energy, it is termed the direct piezoelectric effect. Conversely, when electrical energy induces mechanical deformation in a piezoelectric material, it is referred to as the inverse piezoelectric effect.^[^ ^40^ ^]^ When piezoelectric materials are polarized by ultrasound, the separation of e^−^–h^+^ pairs create an internal electric field. The separated electrons and holes migrate to opposite surfaces, generating a piezoelectric potential that can drive redox reactions.^[^ ^41^, ^42^ ^]^ Taking the inorganic material ZnO as an example, ZnO adopts a hexagonal wurtzite crystal structure. The absence of a symmetric center and the presence of sp^3^ hybridization between zinc and oxygen, forming four equivalent atomic orbitals, are crucial for its piezoelectric and pyroelectric properties (**Figure** **3**a).^[^ ^43^, ^44^ ^]^ When an externally applied force acts on the electrically neutral tetrahedral structure, the positive and negative charge centers shift, resulting in polarization. This results in the accumulation of charges with opposite signs along the direction of stress (Figure 3b).^[^ ^45^, ^46^ ^]^

ZnO, renowned for its high biocompatibility and approved by the U.S. Food and Drug Administration (FDA) as a safe pharmaceutical, is increasingly gaining attention in biomedical research.^[^ ^47^ ^]^ When used as a sonosensitizers, ZnO exhibits SL upon exposure to ultrasound stimulation. The light emitted during SL induces the generation of e^−^–h^+^ pairs in ZnO, triggering redox reactions that produce ROS (Figure 3c).^[^ ^21^, ^48^ ^]^ However, the rapid recombination of e^−^–h^+^ pairs in pure ZnO has been identified as a limiting factor affecting its therapeutic efficacy.^[^ ^49^ ^]^ Consequently, researchers have explored various strategies to modify the electronic structure, bandgap, surface area, and catalytic performance of ZnO by incorporating approaches such as loading with biochar,^[^ ^50^, ^51^, ^52^ ^]^ metal doping,^[^ ^21^, ^53^, ^54^ ^]^ and introducing structural defects.^[^ ^21^, ^54^ ^]^ These measures have significantly enhanced the yield of ROS, as illustrated in Figure 3d.

Interestingly, sonosensitizers, with ZnO as a representative, demonstrate exceptional characteristics as inorganic piezoelectric materials. Upon ultrasound excitation, the crystal surface exhibits positive and negative charges, fulfilling dual roles of electrical stimulation and ultrasound sensitization. Therefore, theoretically, any material capable of generating charges following ultrasound excitation holds potential as an ultrasound sensitizer. **Table** **1** provides a statistical overview of the current applications of piezoelectric materials in SDT.

**Table 1:** Piezoelectric materials sed as Ultrasound Sensitizers.

Piezoelectric Materials	Types of Materials	Material Structures	Piezoelectric Sonosensitizers	Notes	Advantages	Disadvantages
ZnO	Synthesis of Non‐Ferroelectric Ceramics	Chalcopyrite	ZnO^[^ ^58^ ^]^ Mn‐ZnO^[^ ^60^ ^]^ X‐ZnO^[^ ^53^ ^]^	X Represents Metal Particles: Such as Au/Fe^3+^	High melting point, simple preparation, and low deposition temperature	Its piezoelectric properties are relatively weak, often requiring doping with other materials to enhance them
BaTiO_3_	Synthesis of Ferroelectric Ceramics	Perovskite	BaTiO3^[^ ^72^, ^74^ ^]^ X‐BTNPs^[^ ^76^, ^77^ ^]^	X Represents Coupling Agents, Such as TMA/Ab	Exhibits significant ferroelectric, piezoelectric, and pyroelectric properties; possesses photoluminescence and photocatalytic activity	Poor biocompatibility; lack of flexibility.
Poly(vinylidene Fluoride‐Trifluoroethylene) (P(VDF‐TrFE))	Synthesis of Ferroelectric Polymer	Polymer (Semi‐crystalline)	P(VDF‐TrFE)^[^ ^93^ ^]^ D‐P(VDF‐TrFE)^[^ ^23^, ^94^ ^]^	D Represents Imaging or Drug Loading	Easy preparation, flexibility, and good biocompatibility	Poor stability, with low piezoelectric constants and electromechanical coupling coefficients.
Polyvinylidene fluoride (PVDF)	Synthesis of Ferroelectric Polymer	Polymer (Semi‐crystalline)	PVDF film^[^ ^102^ ^]^	Potential Piezoelectric Sensitizers	Exhibits excellent dielectric, ferroelectric, piezoelectric, and pyroelectric properties	Demanding preparation processes.
Poly‐L‐Lactic Acid (PLLA)	Synthesis of Non‐Ferroelectric Polymer	Polymer (Semi‐crystalline)	PLLA^[^ ^150^ ^]^	Undergoing Further Enhancement of Piezoelectric Performance, Potential Piezoelectric Sensitizers	Biodegradable; exhibits good compatibility	Low piezoelectric performance, requiring special treatment to manifest.
BP	2D Semiconducto	Monoelemental Material	BP^[^ ^125^ ^]^ X‐BP^[^ ^22^, ^126^ ^]^	Potential Piezoelectric Materials for Use in Sonodynamic Therapy	Possesses high theoretical specific capacity and electronic conductivity	Low piezoelectric properties, often necessitating doping with other materials for improvement.
Gallium Nitride (GaN)	Synthesis of Non‐Ferroelectric Crystal	Wurtzite	GaN^[^ ^136^, ^137^ ^]^	Used as a Photosensitizer, Potential Piezoelectric Sensitizer	High electron mobility	Higher production costs and relatively complex manufacturing techniques.
Boron Nitride (BN)	Synthesis of Non‐Ferroelectric Crystal	Wurtzite	BN^[^ ^130^, ^133^ ^]^	Acoustic‐Induced Luminescent Characteristics, Potential Piezoelectric Sensitizer	Excellent insulating properties, thermal stability, and optical characteristics, along with good chemical stability	Complex and challenging preparation processes.

## 4 Piezoelectric Sonosensitizers – ZnO

**Figure 4:**
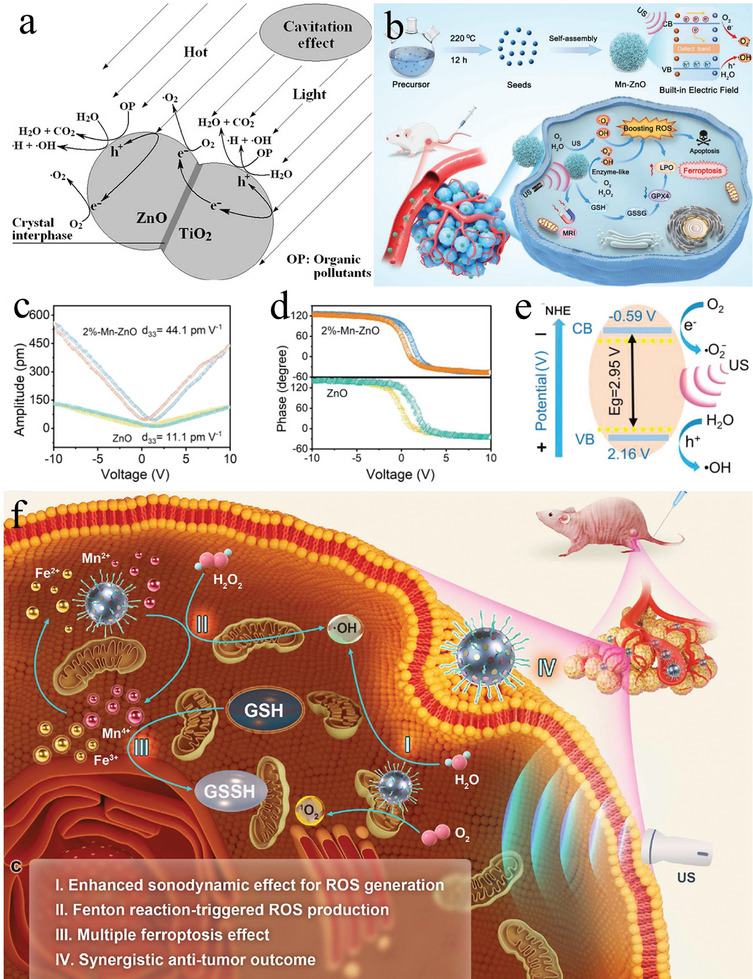
a) Possible principle on excitation of composite TiO_2_/ZnO particle under ultrasonic irradiation^[^ ^57^ ^]^ Copyright 2008 Elsevier B.V. b) Schematic illustration of mesoporous Mn–ZnO nanocluster and its anticancer mechanism of enhanced piezocatalytic therapy and ferroptosis^[^ ^60^ ^]^ Copyright 2023 Wiley‐VCH. c) Amplitude–voltage curves and d) phase hysteresis loops of ZnO and Mn–ZnO^[^ ^60^ ^]^ Copyright 2023 Wiley‐VCH. e) Schematic illustrations on the generation of ROS from Zn–MnO under US irradiation^[^ ^60^ ^]^ Copyright 2023 Wiley‐VCH. f) Schematic illustration of the synthesis of D‐ZnO‐PEG nanosonosensitizers and the ferroptosis‐augmented SDT anti‐tumor effect^[^ ^53^ ^]^ Copyright 2022 The Authors.

ZnO is a prototypical piezoelectric material and has been utilized as a piezoelectric transducer in ultrasound probes as early as 1996.^[^ ^55^ ^]^ In 2008, Wu et al.^[^ ^56^ ^]^ evaluated the degradation effect of ultrasound‐excited UV/ZnO on Reactive Red 198 (RR198). They verified that the generation rates of holes and free radicals increased with the ZnO concentration, suggesting that the integration of ultrasound irradiation with the UV/ZnO system effectively decolorized and mineralized RR198, albeit with limited catalytic efficiency. Subsequently, Wang et al.^[^ ^57^ ^]^ devised a TiO_2_/ZnO composite sono‐catalyst. Under ultrasound action, this composite exhibited superior efficiency in decomposing Acid Red B compared to individual components. Due to differing adsorption tendencies, TiO_2_ favored hole oxidation, whereas ZnO favored free radical oxidation, enabling the complete separation of holes and electrons for enhanced degradation (**Figure** **4**a). In 2009, Perelshtein et al.^[^ ^58^ ^]^ discovered that ZnO thin film coatings could generate free radicals upon ultrasound excitation, exhibiting exceptional antibacterial activity against Escherichia coli and Staphylococcus aureus. This study heralded a groundbreaking application of ZnO in the biomedical domain. ZnO nanoparticles augmented ultrasound‐induced lipid peroxidation, undergoing continuous redox reactions on their surface to generate abundant ROS. Furthermore, ZnO nanoparticles induced cytotoxicity in a cell‐specific and proliferation‐dependent manner, particularly targeting rapidly dividing cells.^[^ ^59^ ^]^

In recent years, the catalytic performance of ZnO nanoparticles has been significantly improved through doping with various metal nanoparticles. Tian et al.^[^ ^60^ ^]^ incorporated porous Mn particles into ZnO (Figure 4b), inducing lattice modifications that increased polarization and enhanced the piezoelectric properties of ZnO nanoparticles (Figure 4c,d). This alteration suppressed the recombination of e^−^–h^+^ pairs at oxygen vacancies, resulting in efficient ROS generation (Figure 4e). Consequently, Mn‐ZnO accelerated the accumulation of lipid peroxides and triggered ferroptosis. Hu et al.^[^ ^53^ ^]^ synthesized D‐ZnO by doping with Fe^3+^ and Mn^4+^ ions. These ions not only depleted glutathione but also initiated a Fenton‐like reaction under the condition of overexpressed H_2_O_2_ in the tumor microenvironment (TME), leading to ferroptosis and enhancing the SDT efficacy of ZnO (Figure 4f). Wang et al.^[^ ^61^ ^]^ bridged Au nanoparticles and ZnO nanoparticles on graphene nanosheets (rGO). Due to rGO's narrow bandgap, e^−^‐ h^+^ pairs generated by ultrasound‐excited ZnO nanoparticles efficiently transferred to Au nanoparticles under the influence of rGO nanosheets. This transfer effectively inhibited the recombination of e^−^–h^+^ pairs, leading to a significant cytotoxic effect on cancer cells. ZnO's non‐centrosymmetric wurtzite structure, characterized by alternating planes of tetrahedrally coordinated Zn^2^⁺ and O^2^⁻, results in charge separation upon lattice distortion induced by external forces. However, ZnO exhibits relatively weak piezoelectric properties, often necessitating doping with other materials to augment its piezoelectric performance.^[^ ^62^ ^]^ Current research has integrated ultra‐hard nanodiamond particles into ZnO through cold sintering technology, achieving prominent nonlinear current‐voltage responses at low voltages by modulating the internal stress within the ZnO matrix.^[^ ^63^ ^]^

ZnO inherently possesses low piezoelectric efficiency, which limits its ability to catalyze ROS production under ultrasonic excitation. The rapid recombination of e^−^–h^+^ pairs in ZnO nanoparticles can be mitigated by the incorporation of metallic nanoparticles. This strategy enhances the piezoelectric potential energy and generates sufficient ROS through redox reactions. Consequently, a prevalent trend in the development of such piezoelectric sonosensitizers involves employing various treatments to decelerate or diminish the swift recombination of e^−^–h^+^ pairs in ZnO nanoparticles.

## 5 Piezoelectric Sonosensitizers – BaTiO_3_

BaTiO_3_, a ferroelectric perovskite material, exhibited excellent piezoelectric properties as early as 1970.^[^ ^64^ ^]^ Initially, BaTiO_3_ found applications in the biomedical field, specifically in inducing neural cell differentiation,^[^ ^65^, ^66^ ^]^ enhancing bone growth,^[^ ^67^, ^68^, ^69^ ^]^ promoting wound healing,^[^ ^70^ ^]^ and regulating early skeletal muscle development.^[^ ^71^ ^]^ In recent years, BaTiO_3_ has emerged as an outstanding sonosensitizers. Under ultrasound stimulation, it generates a powerful intrinsic electric field within its molecules, catalyzing the in‐situ eradication of toxic hydroxyl radicals (·OH) and·O_2_^−^ for tumor removal.^[^ ^72^, ^73^ ^]^ BaTiO_3_ possesses a perovskite structure, with its unit cell comprising one barium ion (Ba^2^⁺), one titanium ion (Ti⁴⁺), and O^2^⁻ positioned at the face centers. The titanium ion is situated at the center of the unit cell, whereas the barium ion occupies eight surrounding positions. This arrangement contributes to the formation of a stable crystal structure. When external mechanical stress is applied to BaTiO_3_, the ions within the crystal undergo displacement, leading to the separation of positive and negative charge centers within the particle, thereby generating charge separation.^[^ ^72^ ^]^

**Figure 5:**
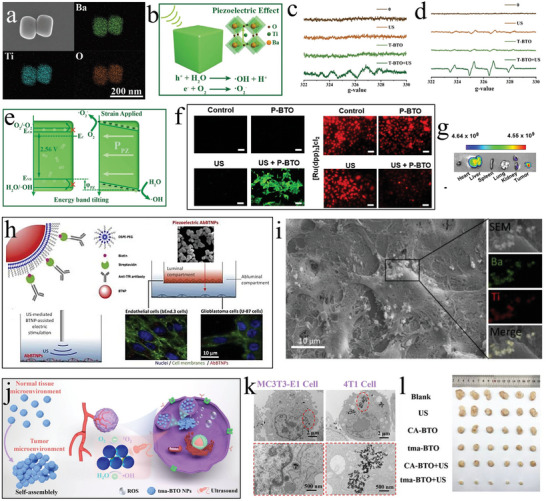
a) STEM image and the corresponding elemental mapping images of T‐BTO^[^ ^72^ ^]^ Copyright 2020 WILEY‐VCH. b) Schematic diagram of the T‐BTO nanoparticles‐mediated piezoelectric effect for the generation of ·OH and ·O_2_^[^ ^72^ ^]^ Copyright 2020 WILEY‐VCH. c,d) ESR spectra of ·O_2_^−^ (c) and ·OH (d) trapped by DMPO in methanol and water respectively under US irradiation^[^ ^72^ ^]^ Copyright 2020 WILEY‐VCH. e) Schematic illustration of band tilting of T‐BTO nanoparticle under applied strain by US‐driven microscopic pressure and the accompanied piezocatalytic redox reaction^[^ ^72^ ^]^ Copyright 2020 WILEY‐VCH. f) Fluorescence images of DCFH‐DA and [Ru(dpp)_3_]Cl_2_in 4T1 cells after different treatments (scale bar: 20 µm)^[^ ^74^ ^]^ Copyright 2021 American Chemical Society. g) Fluorescence imaging of organs collected at 4 h after intravenous injection of Cy5.5 labeled P‐BTO nanoparticles^[^ ^74^ ^]^ Copyright 2021 American Chemical Society. h) the synthesis process of AbBTNPs, a blood‐brain barrier penetration model, and a schematic diagram of chronic piezoelectric stimulation for glioblastoma^[^ ^75^ ^]^ Copyright 2023 Wiley‐VCH. i) SEM imaging and EDX analysis of BTNPs associated to the plasma membranes of bEnd.3 cells (Ba in green and Ti in red)^[^ ^75^ ^]^ Copyright 2023 Wiley‐VCH. j) Schematic illustration of enhanced piezocatalytic performance based on self‐assembly tma‐BTO NPs under US stimuli^[^ ^77^ ^]^ Copyright 2023 The Authors. k) TEM images showing the intracellular location of tma‐BTO aggregations^[^ ^77^ ^]^ Copyright 2023 The Authors. l) solid tumors on day 24^[^ ^77^ ^]^ Copyright 2023 The Authors.

Zhu et al.^[^ ^72^ ^]^ first confirmed the elemental composition of BaTiO_3_ nanoparticles (**Figure** **5**a). After ultrasound stimulation, the internal separation of BaTiO_3_ nanoparticles produced e^−^–h^+^ pairs, and the generation process of ROS (·OH, H^+^, ·O_2_^−^) was illustrated. The micro‐pressure changes inside the crystal and the band tilting during the piezoelectric catalytic redox reaction were also depicted. Subsequently, it was experimentally confirmed in vitro that ultrasound‐stimulated BaTiO_3_ nanoparticles could effectively decompose 5,5‐Dimethyl‐1‐pyrroline N‐oxide (DMPO) to produce ROS (Figure 5b–e). This therapeutic approach was termed piezoelectric catalytic therapy or piezo dynamic therapy. Wang et al.^[^ ^74^ ^]^ developed ultra‐small barium titanate nanoparticles coated with DSPE‐PEG2000, with a particle size of only 6.83 ± 1.75 nm. After ultrasound stimulation, these nanoparticles significantly depleted cellular oxygen content and increased ROS levels (Figure 5f). The coated nanoparticles exhibited excellent biocompatibility and primarily accumulated in the tumor region, as well as the mouse liver and kidneys (Figure 5g). The team further coated BaTiO_3_ with cell membrane‐expressed matrix metallopeptidase 2, significantly enhancing the nanoparticle's accumulation in tumor tissues. While generating ROS, this approach promoted the intratumoral infiltration of cytotoxic T lymphocytes, thereby enhancing the immunotherapy of tumor cells.^[^ ^75^ ^]^ Marino et al.^[^ ^76^ ^]^ functionalized BaTiO_3_ nanoparticles with anti‐transferrin receptor antibodies (anti‐TfR Ab), creating antibody‐functionalized BaTiO_3_ nanoparticles (AbBTNPs) capable of penetrating the blood‐brain barrier and targeting glioblastoma cells simultaneously (Figure 5i). Chronic electrical stimulation induced by ultrasound significantly reduced glioblastoma proliferation (Figure 5h), and the combination with chemotherapy drug temozolomide substantially increased chemotherapy sensitivity. These studies robustly confirmed that individual BaTiO_3_ nanoparticles could generate sufficient ROS for the treatment of tumor cells after ultrasound stimulation. Xiang et al.^[^ ^77^ ^]^ prepared TME‐responsive self‐assembled BaTiO_3_ nanoparticles (tma‐BTO NPs) based on the acidic tumor environment. With an average particle size of 18.15 ± 3.64 nm, these nanoparticles could pass through cell gaps. Stimulated by the TME, they underwent self‐assembly to form submicron aggregates. Compared to individual BaTiO_3_ nanoparticles, the self‐assembled BaTiO_3_ significantly enhanced piezoelectric catalytic efficiency. In in vivo experiments further confirmed that self‐assembled BaTiO_3_ could significantly inhibit tumor growth after ultrasound stimulation (Figure 5j–l).

**Figure 6:**
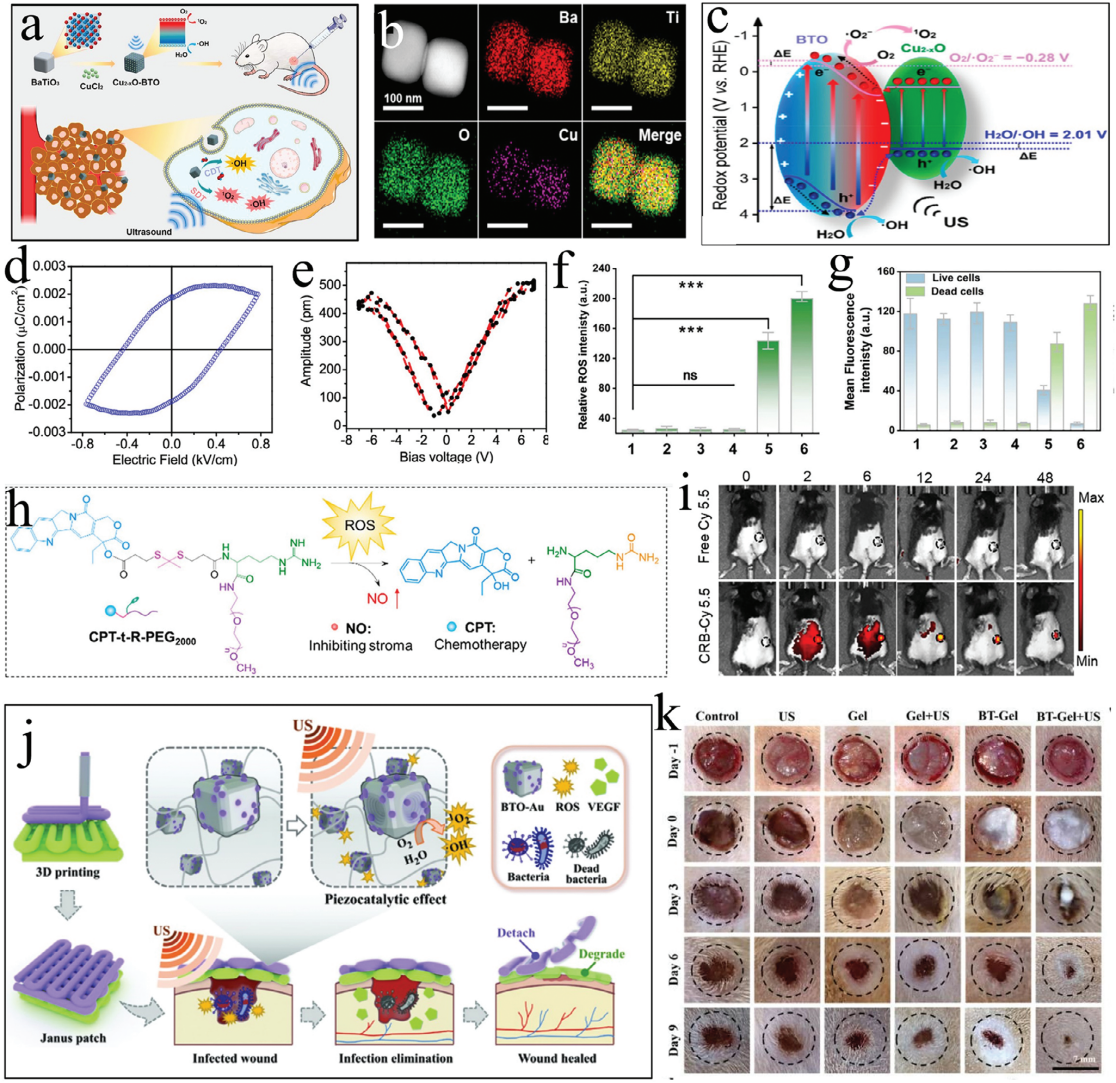
a) Schematic Diagram of the Enhanced Sonodynamic and Chemodynamic Therapy Based on Cu_2–x_O–BTO NCs^[^ ^73^ ^]^ Copyright 2022 American Chemical Society. b) EDX element mapping of Cu_2–x_O–BTO‐1.0^[^ ^73^ ^]^ Copyright 2022 American Chemical Society. c) Schematic diagram of the separation and migration of electron–hole pairs in Cu_2–x_O–BTO NCs with a piezotronic effect^[^ ^73^ ^]^ Copyright 2022 American Chemical Society. d,e) Polarization versus electric field curve and amplitude versus bias voltage plot of the TiO_2_–BaTiO_3_ nanorod film measured at room temperature showing the ferroelectric and piezoelectric property^[^ ^78^ ^]^ Copyright 2020 American Chemical Society. f) Quantitative data of intracellular ROS level in 4T1 cancer cells stained with DCFH‐DA^[^ ^80^ ^]^ Copyright 2023 Wiley‐VCH. g) Quantitative data of Calcein AM/PI staining of 4T1 cells after different treatments^[^ ^80^ ^]^ Copyright 2023 Wiley‐VCH. h) Schematic illustration of the effective tumor chemotherapy with inhibiting stroma and enhancing CPT penetration through US‐triggered piezocatalysis and nanoprodrug strategy^[^ ^81^ ^]^ Copyright 2023 American Chemical Society. i) Real‐time fluorescence images of free Cy5.5 and Cy5.5‐labeled CRB in living mice^[^ ^81^ ^]^ Copyright 2023 American Chemical Society. j) Schemes of the fabrication and application of the Janus patch. The piezoelectric materials and growth‐factor‐coloaded Janus hydrogel patch realized the US‐excited bacteria elimination and promoted wound healing^[^ ^84^ ^]^ Copyright 2023 The Author. k) Representative photographs of infected wounds at different times during the treatment. Scale bars: 7 mm^[^ ^70^ ^]^ Copyright 2022 Elsevier B.V.

In summary, pure BaTiO_3_ nanoparticles have demonstrated excellent ultrasound sensitizing properties, generating sufficient ROS under ultrasound excitation for tumor cell elimination. Zhao et al.^[^ ^73^ ^]^ synthesized piezoelectric composite nanoparticles based on BaTiO_3_ (Cu_2‐x_O‐BaTiO_3_) using a simple two‐step method. Under ultrasound excitation, effective electron separation occurred in BaTiO_3_, and due to the lower energy levels of Cu_2‐x_O, e^−^‐h^+^ pairs in BaTiO_3_ tended to migrate to Cu_2‐x_O. The continuous action of ultrasound radiation led to an expanding potential difference between the energy levels and redox potentials, thereby increasing ROS production (**Figure** **6**a–c). The authors proposed that Cu_2‐x_O‐BaTiO_3_ piezoelectric nanoparticles, as sensitizers, could enhance the efficacy of cancer treatment.

Some researchers^[^ ^78^ ^]^ employed BaTiO_3_ as the substrate and synthesized TiO_2_–BaTiO_3_ nanorods through the addition of TiO_2_ sonosensitizers. This resulted in exceptional piezoelectric performance (Figure 6d,e) and achieved remarkable piezoelectric catalytic efficiency. Wang et al.^[^ ^79^ ^]^ coated few‐layer MoS_2_ nanosheets onto BaTiO_3_ and modified them with pH‐responsive cinnamic acid. Under ultrasound excitation, this led to enhanced peroxidase‐like activity and glutathione depletion. Deng et al.^[^ ^80^ ^]^ introduced palladium nanoparticles to impart dual enzyme‐mimicking properties to piezoelectric composite nanoparticles. During the synthesis process, the bandgap of platinum was reduced, slowing down the recombination rate of e^−^–h^+^ pairs and achieving high ROS production (Figure 6f,g).

Wang et al.^[^ ^81^ ^]^ covalently coupled camptothecin (CPT) and L‐arginine through sulfurized Schiff base bonds on the BaTiO_3_ substrate. Following ultrasound stimulation, ROS generated by BaTiO_3_ caused the cleavage of sulfur bonds, releasing CPT, and oxidizing L‐arginine to produce nitric oxide (NO) molecules. This achieved targeted drug delivery and enhanced CPT chemotherapy sensitivity (Figure 6h,i). Zhao et al.^[^ ^82^ ^]^ incorporated L‐arginine into strontium‐doped (Sr) BaTiO_3_ piezoelectric nanoparticles, obtaining BST@LA piezoelectric nanoparticles as sensitizers. Under ultrasound excitation, these nanoparticles produced NO and exhibited a significant tumor inhibition rate (89.5%) in 4T1 tumor‐bearing mice. Additionally, piezoelectric films^[^ ^83^ ^]^ or gels^[^ ^70^, ^84^, ^85^ ^]^ based on BaTiO_3_, when stimulated by ultrasound, exhibited excellent antibacterial effects and promoted wound healing, as shown in Figure 6j,k. Furthermore, piezoelectric sensitizers prepared from BaTiO_3_ could promote bone tissue growth during the anti‐inflammatory process.^[^ ^20^, ^86^ ^]^

BaTiO₃, as an exceptional inorganic piezoelectric material, possesses the capability to generate a potent internal electric field within its molecules under ultrasonic stimulation. This, in turn, produces sufficient ROS for tumor treatment. However, despite being improvable through coating techniques, its poor biocompatibility poses significant challenges for clinical translation. To address this, piezoelectric composite nanoparticles have been constructed by incorporating other particles into BaTiO₃ as the base material. These composites can further enhance ROS generation. The underlying mechanism may involve the transfer of electron separation generated by BaTiO₃ to nanoparticles with lower energy levels, thereby increasing the potential energy difference between the energy band levels and the redox potential of BaTiO₃. This, in effect, leads to the production of more ROS. Therefore, sonodynamic therapy based on barium titanate holds great promise for advancements in tumor research.

## 6 Piezoelectric Sonosensitizers – P(VDF‐TrFE) and PVDF

**Figure 7:**
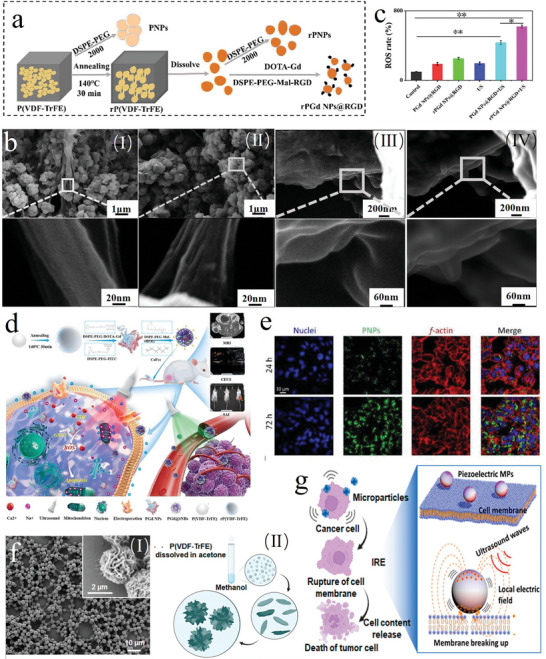
a) Schematic diagram of rPGd NPs@RGD composite piezoelectric nanoparticle synthesis^[^ ^23^ ^]^ Copyright 2023 American Chemical Society. b) SEM image of rP(VDF‐TrFE) and P(VDF‐TrFE) nanoparticles: (I) SEM of rP(VDF‐TrFE) nanoparticles; (II) SEM of P(VDF‐TrFE) nanoparticles; (III) Amorphous region of P(VDF‐TrFE) nanoparticles, before recrystallization; (IV) Amorphous region of P(VDF‐TrFE) nanoparticles, after recrystallization^[^ ^23^ ^]^ Copyright 2023 American Chemical Society. c) Quantification of ROS generation^[^ ^23^ ^]^ Copyright 2023 American Chemical Society. d) Schematic diagram of the synthetic process and antitumor mechanism of PGd@tNBs^[^ ^95^ ^]^ Copyright 2024 The Author(s). e) Internalization of fluorescently‐labeled PNPs in T98G cells at 24 and 72 h of incubation. Confocal fluorescence microscopy imaging of nuclei (blue), DiO‐stained PNPs (green) and f‐actin (red)^[^ ^94^ ^]^ Copyright 2021 The Authors. f) SEM image of a representative as‐received P(VDF‐TrFE) MP supported on a Si substrate. Inset: I) Enlarged SEM image showing the MPs surface texture; II) Schematic showing the MP growth mechanism via Ostwald ripening^[^ ^93^ ^]^ Copyright 2023 American Chemical Society. g) Schematic showing P(VDF‐TrFE) cancer cell killing mechanism via ultrasound‐mediated IRE effect^[^ ^93^ ^]^ Copyright 2023 American Chemical Society.

P(VDF‐TrFE) is a semi‐crystalline ferroelectric polymer characterized by a mixture of crystalline (ordered) and amorphous (unordered) regions.^[^ ^87^ ^]^ This polymer exhibits a spectrum of crystalline phases, including α, β, γ, δ, and ε, with the β phase distinguished by its superior ferroelectric and piezoelectric properties. In the β phase, fluorine and hydrogen atoms are positioned on opposite sides of the carbon chain, generating a dipole moment. The dipoles within each unit cell align uniformly, resulting in a robust polarity. Upon exposure to ultrasonic stimulation, polarized charges are produced, leading to the exhibition of piezoelectric potentials.^[^ ^88^, ^89^ ^]^ In recent years, the utilization of P(VDF‐TrFE) piezoelectric materials in the biomedical field has increased significantly. Through external ultrasound stimulation, P(VDF‐TrFE) piezoelectric films have been shown to enhance neuronal differentiation,^[^ ^90^ ^]^ polarization macrophages,^[^ ^91^ ^]^ induce osteogenic differentiation of cells,^[^ ^92^ ^]^ promote wound healing,^[^ ^83^ ^]^ and affect cell proliferation, migration, and even apoptosis.^[^ ^23^, ^93^, ^94^ ^]^ Our team^[^ ^23^ ^]^ conducted in‐depth research on P(VDF‐TrFE) piezoelectric nanoparticles. By encapsulating them with polyethylene glycol, we effectively improved the hydrophilicity of P(VDF‐TrFE). Furthermore, we discovered that high‐temperature crystalline reshaping enhanced the compliance of unordered convolution areas in the amorphous region of the nanoparticles. We hypothesize that unordered convolution areas represent a macroscopic manifestation of the polymer chain in P(VDF‐TrFE) nanoparticles. By revealing changes in microscopic morphology, we uncovered the potential mechanism by which P(VDF‐TrFE)‐based piezoelectric composite nanoparticles enhance SDT through ultrasound‐activated piezoelectric materials generating chronic electrical stimulation and augmenting the production of ROS (**Figure** **7**a–c). Importantly, we have also found that piezoelectric composite nanoparticles constructed based on P(VDF‐TrFE) exhibit exceptional therapeutic and imaging properties. Specifically, the piezoelectric electrical stimulation excited by ultrasound sensitizes the treatment of HER2‐positive breast cancer (Figure 7d).^[^ ^95^ ^]^

Pucci et al.^[^ ^94^ ^]^ loaded nutlin‐3a onto ApoE‐functionalized P(VDF‐TrFE) nanoparticles, which could penetrate the blood‐brain barrier, efficiently accumulate in glioma cells, and, upon ultrasound stimulation, influence cell proliferation and induce cell death through localized electrical stimulation (Figure 7e). These nanoparticles also demonstrated anti‐angiogenic effects, impacting renal tubular vessel formation and disruption.^[^ ^96^ ^]^ Silva et al.^[^ ^93^ ^]^ prepared P(VDF‐TrFE) particles that, under ultrasound stimulation, could eliminate 4T1 breast cancer cells through irreversible electroporation (Figure 7f,g). While P(VDF‐TrFE) piezoelectric materials exhibit excellent biocompatibility, their piezoelectric performance is significantly lower than that of inorganic piezoelectric materials such as BaTiO_3_. After ultrasound excitation, the charge distribution on the material surface is not sufficient to cause irreversible electroporation of the cell membrane, resulting in reversible electroporation. Consequently, the generated charge can directly act on the cell membrane or increase the production of ROS through redox reactions to damage tumor cells.

PVDF is another semi‐crystalline ferroelectric polymer with a high piezoelectric coefficient (d_33_ = 49.6 pm/V). It is widely used in various fields, including sensors, transducers, optical devices, and biomaterial scaffolds.^[^ ^97^ ^]^ However, its application in the biomedical field has been relatively limited, primarily for regulating the proliferation and differentiation of neural stem cells,^[^ ^98^ ^]^ enhancing cell adhesion,^[^ ^99^ ^]^ promoting macrophage polarization,^[^ ^100^ ^]^ and inhibiting bacterial growth.^[^ ^101^ ^]^ There is limited research on the use of PVDF as a sonosensitizer with only one report^[^ ^102^ ^]^ mentioning the embedding of hydroxyapatite nanowires into PVDF films. Under ultrasound excitation, the cell membrane undergoes reversible electroporation, achieving the purpose of drug delivery.

P(VDF‐TrFE), as an organic semi‐crystalline ferroelectric polymer, exhibits excellent biosafety but has piezoelectric properties influenced by the β‐phase content, generally lower than those of inorganic piezoelectric materials such as BaTiO3. Currently, there is a lack of in‐depth research on the generation of ROS in piezoelectric composite nanoparticles synthesized using P(VDF‐TrFE) materials. Therefore, future studies should explore the addition of nanoparticles with lower energy bands to P(VDF‐TrFE) to investigate changes in the polymer's energy band and the generation of ROS after ultrasonic excitation.

## 7 Piezoelectric Sonosensitizers – Natural Piezoelectric Biomaterials

**Figure 8:**
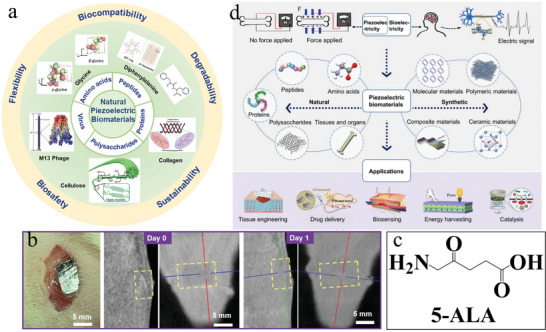
a) Schematic of the five major types of natural piezoelectric biomaterials and their properties for biomedical applications^[^ ^103^ ^]^ Copyright 2022 American Chemical Society. b) Observation of the biodegradation of a glycine‐PVA film inside a rat body at the subdermal dorsal region. The left image shows the device being implanted; the middle and right images are CT images of the implantation area immediately and 1 day after implantation, respectively. Yellow boxes mark the location of the implanted film^[^ ^111^ ^]^ Copyright 2022 The Author. c) Molecular formula of 5‐ALA. d) Bio‐inspired piezoelectric materials for applications in biomedicine and nanotechnology: from bioelectricity, natural and synthetic materials, to various applications^[^ ^124^ ^]^ Copyright 2024 Wiley‐VCH.

Natural piezoelectric materials encompass substances from biological systems, boasting inherent flexibility, exceptional biocompatibility, and near‐complete degradability. These materials encompass amino acids, peptides, and their polymeric forms such as proteins and polysaccharides (**Figure** **8**a).^[^ ^103^, ^104^ ^]^ Among the 20 amino acids in natural proteins, 17‐excluding asparagine, phenylalanine, and tryptophan‐demonstrate piezoelectricity.^[^ ^105^, ^106^ ^]^ Amino acids exhibit a broad range of piezoelectric coefficients, spanning from 0.5 pC/N to 178 pC/N. Notably, β‐glycine crystals exhibit a high shear piezoelectric coefficients of up to 178 pm V^−1^ upon supramolecular stacking, comparable to inorganic piezoelectric materials like barium titanate or lead zirconate titanate.^[^ ^107^ ^]^ Furthermore, they surpass the maximum piezoelectric coefficients of other biological piezoelectric materials, including, γ‐glycine,^[^ ^108^ ^]^ PVDF polymers,^[^ ^109^ ^]^ and peptide nanotubes,^[^ ^110^ ^]^ and demonstrate outstanding biocompatibility (Figure 8b).^[^ ^111^ ^]^ 5‐Aminolevulinic acid (5‐ALA) is a non‐protein amino acid ubiquitous in animals and plants, serving as a precursor for the biosynthesis of all porphyrin compounds.^[^ ^112^ ^]^ Research has established that 5‐ALA, acting as a sonosensitizer, can enhance the inactivation of various tumor cells, including breast cancer cells,^[^ ^112^, ^113^ ^]^ malignant glioma cells,^[^ ^114^ ^]^ melanoma cells,^[^ ^115^ ^]^ pancreatic cancer cells,^[^ ^116^ ^]^ human tongue squamous cell carcinoma,^[^ ^117^ ^]^ and human colon cancer cells.^[^ ^118^ ^]^ The asymmetric arrangement within the 5‐ALA molecule suggests its potential as a piezoelectric material (Figure 8c). Under high‐energy shock waves, it augments intracellular ROS generation, causing damage to colon or thyroid cancer cells.^[^ ^118^, ^119^ ^]^

Peptide chains, consisting of short sequences of amino acids, share numerous structural and property similarities with amino acids, such as phenylalanine (FF),^[^ ^120^ ^]^ and cyclo‐glycine‐tryptophan (cyclo‐GW).^[^ ^121^ ^]^ Fmoc FF and cyclo GW exhibit the highest piezoelectric coefficients of d15‐33.7 pm/V and d16‐14 pC/N, respectively.^[^ ^122^ ^]^ In contrast, Proteins are intricate macromolecules composed of multiple amino acids, with their piezoelectricity stemming from the accumulation of dipoles along the peptide chain and the hydrogen bonding of supramolecular stacking.^[^ ^104^ ^]^ In addition to the piezoelectricity exhibited by proteins derived from amino acids, plant‐based polysaccharide materials also demonstrate piezoelectric effects. Cellulose, the most abundant polysaccharide and a key component of plant biomass, consists of fibrous polymeric polysaccharides formed through β‐1,4‐linkages of D‐glucose residues. However, the piezoelectric coefficient values (shear, lateral, and longitudinal) of cellulose samples prepared from various sources and methods display a wide range.^[^ ^123^ ^]^ Notably, the piezoelectric coefficients of natural piezoelectric materials are significantly lower compared to those of inorganic piezoelectric materials. Currently, efforts to enhance the piezoelectricity of biomaterials can be approached from two distinct levels. At the macro level, improvements in piezoelectric performance can be achieved through effective structural optimization designs, such as the fabrication of suitable nanostructures and the construction of multilayer structures. At the molecular level, given that piezoelectric biomaterials typically contain abundant functional groups and possess the ability to bind other ions, their inherent polarization and piezoelectricity can be augmented by modulating the molecular structure of the biomaterials.^[^ ^124^ ^]^

Natural piezoelectric biomaterials exhibit superior biocompatibility and nearly complete degradability, rendering them highly promising for translational applications in human biological tissues. Although the piezoelectricity generated by the supramolecular stacking of β‐glycine crystals can rival that of inorganic piezoelectric materials like barium titanate or lead zirconate titanate, β‐glycine crystals readily transform into α‐glycine at room temperature, drastically diminishing their piezoelectric performance. Consequently, a pivotal focus of future research will be to stabilize the piezoelectric properties of natural piezoelectric biomaterials at room temperature.

## 8 Piezoelectric Sonosensitizers – BP

**Figure 9:**
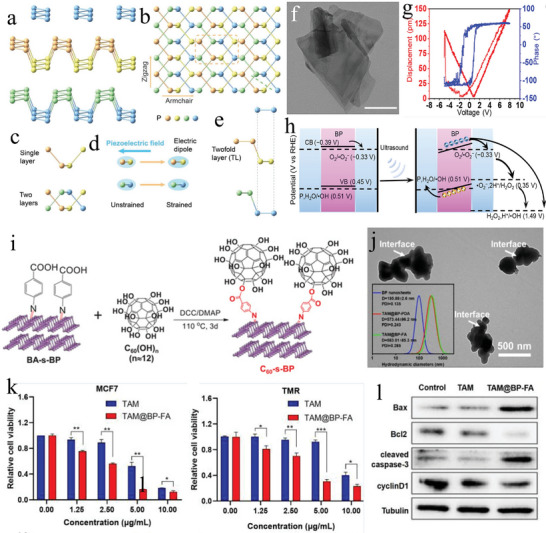
a) Atomic lattice structure of BP, in order to identify the different positions of the P atoms, four colors are used^[^ ^125^ ^]^ Copyright 2020 WILEY‐VCH. b) The top view of the bilayer BP structure^[^ ^125^ ^]^ Copyright 2020 WILEY‐VCH. c) The basic structural unit of the bilayer, which is orange frame in (b)^[^ ^125^ ^]^ Copyright 2020 WILEY‐VCH. d) The piezoelectric dipole and the field which exist in bilayer BP under strain in the armchair direction^[^ ^125^ ^]^ Copyright 2020 WILEY‐VCH. e) The side view of the bilayer BP structure. The viewing direction is shown as a green line in (b). The blue P atoms in layers 1 and 3 correspond to each other. This indicates that the component units of bulk BP are in two layers along the out‐of‐plane direction. Thus, piezoelectric properties can exist in multilayer BP^[^ ^125^ ^]^ Copyright 2020 WILEY‐VCH. f) TEM image of a BP nanosheet. Scale bar = 200 nm.^[^ ^124^ ^]^ g) Height hysteresis loop under different tip–substrate voltages^[^ ^126^ ^]^ Copyright 2020 American Chemical Society. h) Schematic illustrations on (left) the intrinsic energy bands of our BP nanosheet and (right) its tilted energy bands under piezoelectric field excited by ultrasound exposure, which facilitate its production of ·OH^[^ ^126^ ^]^ Copyright 2020 American Chemical Society. i) Scheme of the preparation process and structure of C_60_‐s‐BP^[^ ^127^ ^]^ Copyright 2021 The Authors. j) TEM of TAM@BP‐FA nanocomposites^[^ ^22^ ^]^ Copyright 2023 Wang et al. k) MCF7 and TMR cells exposed to free TAM or TAM@BP‐FA at different concentrations^[^ ^22^ ^]^ Copyright 2023 Wang et al. l) Protein analysis of TMR cells after the above treatments (n = 2)^[^ ^22^ ^]^ Copyright 2023 Wang et al.

BP, a single‐element 2D material, is predicted to exhibit piezoelectric properties due to its highly directional and non‐centrosymmetric crystal lattice structure. Despite limited research reporting its piezoelectric phenomenon due to the absence of ion polarization, theoretical calculations on BP graphene indicate that its piezoelectricity originates from phosphorus atoms. However, in multilayer 2D materials, piezoelectricity diminishes primarily due to opposing orientations between adjacent atomic layers (Figure 9a–e).^[^ ^125^ ^]^

Li et al.^[^ ^126^ ^]^ successfully obtained thin‐layer BP nanosheets through ultrasonic exfoliation of bulk BP, with an average thickness of 5.3 ± 3.7 nm and an average lateral size of 162.4 ± 99.4 nm (Figure 9f). Upon applying an external voltage of 8V, the piezoelectric phase map of BP nanosheets underwent a 180° flip, confirming their excellent piezoelectric properties (Figure 9g). The band gap of BP nanosheets is ≈0.84 eV, with the conduction band (CB) and valence band (VB) edges located at −0.39 and 0.45 V, respectively. The negative voltage along the CB edge slightly exceeds the redox potential of O_2_/·O_2_^−^ (−0.33 V), suggesting that electrons on the CB are favorable for the O_2_/·O_2_^−^ redox reaction. Conversely, the positive voltage at the VB edge is significantly lower than the redox potential of H_2_O/·OH, hindering the direct transfer of hole charges from the VB to water molecules to generate ·OH. Under ultrasonic excitation, BP nanosheets undergo piezoelectric polarization, resulting in the separation of e^−^–h^+^ pairs. This separation tilts the BP band, further increasing the voltage difference between the CB and VB, thereby enhancing the ability of the generated charges to drive ROS production and achieving the goal of tumor treatment (Figure 9h). In subsequent studies, the team^[^ ^127^ ^]^ functionalized BP nanosheets with fullerene to improve stability, biocompatibility, and SDT efficiency against 4T1 cells upon ultrasonic excitation (Figure 9i,j).

Recently, numerous studies have focused on using BP as a base material and enhancing its SDT efficacy by incorporating various therapeutic drugs,^[^ ^22^ ^]^ metal nanoparticles,^[^ ^128^, ^129^ ^]^ and sonosensitizers.^[^ ^130^ ^]^ For example, Wang et al.^[^ ^22^ ^]^ utilized BP as a carrier to combine tamoxifen (TAM) and the tumor‐targeting ligand folic acid (FA), constructing TAM@BP‐FA (Figure 9k) for endocrine therapy and SDT of estrogen receptor‐positive breast cancer. The ROS generated by SDT lowered the mitochondrial membrane potential, causing mitochondrial damage.Additionally, TAM@BP‐FA promoted natural killer (NK) cell infiltration and immune suppression through macrophage depletion, potentially activating an anti‐tumor immune response (Figure 9l). Compared to free TAM, TAM@BP‐FA significantly upregulated the expression of Bax and Caspase‐3 in tumor cells while downregulating Bcl‐2 expression (Figure 9m).

## 9 Piezoelectric Sonosensitizers – Other Piezoelectric Materials

**Figure 10:**
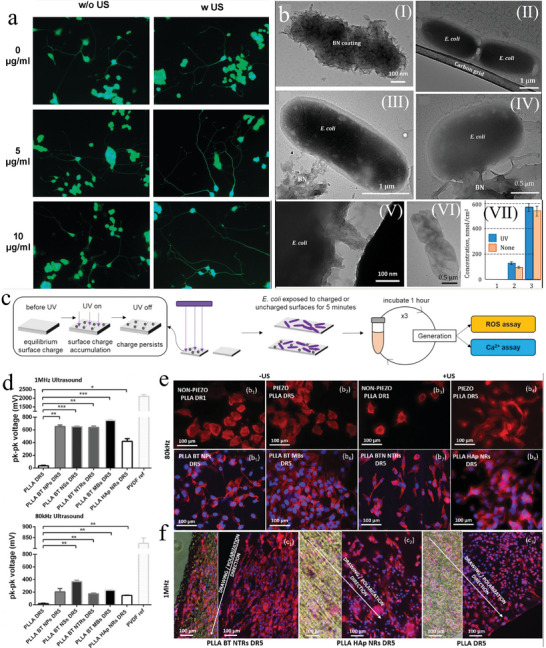
a) Images of calcein‐labeled PC12 cells after 9 days of treatments^[^ ^133^ ^]^ Copyright 2010 American Chemical Society. b) TEM images of a piece of BN coating (I), control E. coli cells (II), and E. coli cells after they were in contact with the BN coating (III–VI). Petal‐like surface of BN coating which pierces (damages) the cell membrane (III–V). Dead cell (VI). Concentration of fluorescein generated in NSS after UV irradiation for 1 h (VII). 1–3 h, 2–8 h, and 3–24 h^[^ ^134^ ^]^ Copyright 2020 American Chemical Society. c) GaN produces ROS and calcium ions to regulate oxidative stress in Escherichia coli after exposure to UV radiation^[^ ^137^ ^]^ Copyright 2020 American Chemical Society. d) Voltage generated by drawn PLLA DR5 films (without or with 1 wt% fillers, including BT NTRs, BT NPs, BT NSs, BT MBs, and HAp NRs) after deformation with 1 MHz and 80 kHz ultrasound and their comparison to standard piezo‐PVDF film. Statistical analysis was performed relative to PLLA (without NPs)^[^ ^151^ ^]^ Copyright 2023 Wiley‐VCH. e) Influence of the piezoelectricity and piezostimulation on cellular shape‐ the cells grown to the surface of nonpiezoelectric PLLA DR1 and piezoelectric PLLA DR5 as well as grown onto filler‐modified PLLA films GaN produces ROS and calcium ions to regulate oxidative stress in Escherichia coli after exposure to UV radiation^[^ ^151^ ^]^ Copyright 2023 Wiley‐VCH. f) Unidirectional elongation and alignment of cells to drawing directions in the case of PLLA NTRs and PLLA HAp NR films. Statistical analysis was performed for actin filament formation per cell on the surface of piezoelectric (DR5) films relative to their nonpiezoelectric (DR1) counterparts^[^ ^151^ ^]^ Copyright 2023 Wiley‐VCH.

BN nanoparticles serve as structural analogs of carbon materials, where carbon atoms are alternately substituted by boron and nitrogen atoms. BN exhibits favorable biocompatibility and minimal cytotoxicity.^[^ ^131^ ^]^ Despite limited research on the therapeutic applications of ultrasound‐excited BN for tumor treatment, considerable attention has been devoted to BN's photoluminescent properties.^[^ ^132^ ^]^ Ciofani et al.,^[^ ^133^ ^]^ through ultrasound excitation BN nanoparticles, facilitated the growth of neuron‐like cell neurites (**Figure** **10**a). Conversely, Gudz et al.^[^ ^134^ ^]^ observed that immersing BN films in physiological saline induced the generation of ROS and efficiently inhibited bacterial growth (Figure 10b). Hence, leveraging BN as a piezoelectric ultrasound sensitizer to produce ROS under ultrasound excitation for tumor treatment is feasible.

GaN is a semiconductor compound distinguished by its exceptional piezoelectric, optoelectronic, and piezoresistive properties. It is widely utilized in thermal biosensors,^[^ ^135^ ^]^ and photothermal therapy,^[^ ^136^ ^]^ among other applications. Notably, under light exposure, GaN surfaces can generate charges and produce ROS, thereby regulating oxidative stress in Escherichia coli (Figure 10c).^[^ ^137^, ^138^ ^]^ Consequently, GaN is recognized as a photosensitizer. However, the majority of sonosensitizers are derived from photosensitizers. GaN's ability to generate charges and produce ROS under light exposure suggests a potential mechanism for SDT to generate ROS. Furthermore, aluminum nitride and lithium niobate are commonly employed as high‐temperature piezoelectric transducers and sensors.^[^ ^139^, ^140^, ^141^ ^]^

PLLA is a crucial biodegradable polymer due to its outstanding biocompatibility and bioabsorbability. It finds extensive application in tissue engineering scaffolds,^[^ ^142^, ^143^ ^]^ medical implants,^[^ ^144^ ^]^ drug delivery systems, and ultrasound imaging.^[^ ^145^, ^146^, ^147^, ^148^, ^149^, ^150^ ^]^ Recently, research has shifted toward PLLA's piezoelectric properties. Vukomanović et al.^[^ ^151^ ^]^ significantly enhanced PLLA's piezoelectric performance by incorporating a low content (1wt%) of high aspect ratio, piezoelectric or non‐piezoelectric filler particles (Figure 10d). Although the ROS produced by ultrasound‐excited modified PLLA films did not increase substantially, they promoted the growth of skin keratinocytes and the formation of cell scaffolds, thereby accelerating wound healing in the biological tissues (Figure 10e,f). Therefore, with further improvements in PLLA's piezoelectric performance, it is anticipated to emerge as a novel sonosensitizer, achieving tumor cells killing under ultrasound exposure.

## 10 Summary and Outlook

SPDT should be considered a specialized branch of SDT, primarily focusing on the utilization of piezoelectric materials as sonosensitizers. Piezoelectric materials that possess sonosensitizing properties are collectively termed piezoelectric sonosensitizers. SDT mediated through these piezoelectric sonosensitizers is referred to as SPDT. The essence of SPDT lies in the separation of e^−^–h^+^ pairs within piezoelectric materials upon ultrasonic excitation. The resultant separated electrons and holes migrate to opposite surfaces of the material, driving redox reactions due to the generated piezoelectric potential. This mechanism leads to the production of ROS for SDT.^[^ ^19^, ^23^, ^42^ ^]^ Traditional sonosensitizers encounter limitations such as low water solubility, inadequate tumor specificity, and susceptibility to metabolism, which hinder their adequate retention within tumor tissues and, consequently, their therapeutic efficacy.^[^ ^9^, ^10^, ^152^ ^]^ Therefore, the identification of novel ultrasonic sensitizers remains a critical bottleneck in the advancement of SDT. Piezoelectric materials, owing to their unique piezoelectric properties, hold immense promise in pressure sensing, piezoelectric catalysis, piezoelectric integration, and energy harvesting.^[^ ^153^ ^]^

Our team's earlier experiments^[^ ^23^ ^]^ revealed that the P(VDF‐TrFE) piezoelectric material, following crystalline reshaping, hydrophilicity enhancement, and imaging integration, successfully achieved SPDT in glioma cells under ultrasonic excitation. This established P(VDF‐TrFE) as a viable sonosensitizer in SDT. A retrospective analysis of ZnO, BaTiO_3_, and natural biological piezoelectric materials systematically outlines their roles in SDT. Enhanced piezoelectric effects were achieved through crystalline structure reshaping,^[^ ^23^ ^]^ defect control,^[^ ^21^, ^54^ ^]^ and targeted modification^[^ ^154^ ^]^ of piezoelectric materials, leading to ultrasound‐responsive SPDT. In our investigation, we observed that after ultrasonic excitation, piezoelectric materials undergo the separation of e^−^–h^+^ pairs within their molecular structure. This separation increases the charge density in the CB and VB, elevating the voltage potential at both ends of the material. Furthermore, when paired with metal nanoparticles,^[^ ^61^ ^]^ the charge transfer to these nanoparticles further augments the piezoelectric potential at both ends of the piezoelectric material. This increased potential facilitates the occurrence of redox reactions. Consequently, any material that generates piezoelectric potential upon ultrasonic excitation can trigger redox reactions is suitable for SPDT. Natural piezoelectric materials, due to their unique biological characteristics, exhibit exceptional safety in vivo. Notably, the piezoelectric coefficient of β‐glycine can reach up to 178 pm V^−1^, which is comparable to inorganic piezoelectric materials such as barium titanate or lead zirconate titanate.^[^ ^107^ ^]^ As a result, the ROS produced upon ultrasonic excitation are sufficient to induce cell damage for therapeutic purposes. Therefore, these materials hold great promise for the development and application of SPDT.

The research and application of SPDT continue to confront several significant challenges. First, inorganic piezoelectric materials exhibit superior piezoelectric properties, yet their clinical translation and application are hampered by poor biocompatibility and high toxicity. Second, organic piezoelectric materials boast exceptional biosafety, but their piezoelectric performance is substantially inferior. Thirdly, natural piezoelectric biomaterials demonstrate remarkable piezoelectric properties under specific conditions, but their stability at room temperature is poor, often resulting in the transformation into less piezoelectric crystalline forms. In light of these shortcomings, future research should prioritize the optimization of piezoelectric materials. The aim should be to enhance their piezoelectric properties while ensuring biocompatibility, thereby addressing these critical limitations.

In piezoelectric materials, the application of mechanical stress results in lattice deformation, which subsequently alters the existing non‐electric field and leads to band bending. This, in turn, triggers the separation and migration of electron‐hole pairs to opposite surfaces of the piezoelectric semiconductor. This bending effect is particularly pronounced at heterojunction interfaces, where differences in electron affinity, work function, and other parameters between adjacent materials create distinct charge accumulation and depletion regions, resulting in a sharp bending of the energy band structure at the interface. Band bending modifies the charge distribution within the material, thereby facilitating the generation and accumulation of polarization charges. When the CB potential of the piezoelectric semiconductor is more negative than the redox potential of O_2_/·O_2_‐ (‐0.33 V), it favors the catalytic production of ·O_2_^−^ from O_2_, potentially also generating 1O2 through energy exchange or cascade reactions. Similarly, when the VB potential is more positive than the oxidation potential of H_2_O/·OH (+2.01 V), it promotes the formation of ·OH.^[^ ^126^ ^]^ This process not only enhances the charge‐stress coupling effect in piezoelectric materials but also allows for effective modulation of the piezoelectric coefficient by adjusting the density and distribution of polarization charges. Furthermore, band bending may alter the conductivity of the material, particularly in the bent regions, influencing the charge transport pathways and efficiency within the material. By enhancing the local charge response speed, it is possible to optimize the output characteristics of piezoelectric signals.^[^ ^155^, ^156^ ^]^

## Conflict of Interest

The authors declare no conflict of interest.

## Author Contributions

C.Z. and B.Z. performed conceptualization. C.Z. performed methodology. B.Z. and L.Y. performed validation. C.Z. wrote the original draft preparation. C.Z., L.X., and B.Z. performed wrote, review and edited the final manuscript. L.Y. performed funding acquisition. All authors have read and agreed to the published version of the manuscript.
